# Fournier’s gangrene caused by penetration of a rectal cancer followed by neoadjuvant chemotherapy

**DOI:** 10.1186/s40792-018-0526-0

**Published:** 2018-09-26

**Authors:** Daigo Kobayashi, Mariko Masubuchi, Tsunenobu Takase, Takahiro Ichikawa, Tomohiro Deguchi, Toyohisa Yaguchi

**Affiliations:** 1 Department of Surgery, Komaki City Hospital, 1-20 Joubushi, Komaki, Aichi 485-8520 Japan; 2 Department of Surgery, Kainan Hospital, 396 Minamihonden, Maegasu-cho, Yatomi, Aichi 498-8502 Japan

**Keywords:** Fournier’s gangrene, Locally advanced rectal cancer, Neoadjuvant chemotherapy

## Abstract

**Background:**

Fournier’s gangrene is a necrotizing fasciitis of the genital and perineal region. It may progress, affecting the groin, the thigh, or even the abdominal wall.

Despite adequate treatment (debridement and antibiotics), the mortality rate is very high, reaching 20–35%. Fournier’s gangrene caused by penetration of a rectal cancer followed by neoadjuvant chemotherapy is very rare. We report this case with a review of the literature.

**Case presentation:**

A 68-year-old man visited the emergency room due to perineal pain during which he accepted the chemotherapy for locally advanced rectal cancer. Abdominal CT scan showed extensive emphysema in the scrotum and gluteus maximus muscle. We diagnosed as Fournier’s gangrene caused by penetration of a rectal cancer. We performed debridement, left orchiectomy, transverse colostomy with double orifices. Post-operative day 30, we performed abdominoperineal resection. We performed CapeOX therapy eight courses as adjuvant chemotherapy. The patient had no recurrence for 1 year and 2 months after the operation.

**Conclusions:**

Going forward, knowledge gained from this case will increase the opportunity to perform neoadjuvant chemotherapy for locally advanced rectal cancer. In medical treatment, we must put the possibility of Fournier’s gangrene in mind and treat as soon as possible.

## Background

Fournier’s gangrene is a necrotizing fasciitis of the genital and perineal region. It may progress, affecting the groin, the thigh, or even the abdominal wall.

Despite adequate treatment (debridement and antibiotics), the mortality rate is very high, reaching 20–35% [1]. There has been no report on the onset of Fournier’s gangrene caused by penetration of a rectal cancer followed by neoadjuvant chemotherapy. We report this case with a review of the literature.

## Case presentation

**Fig. 1:**
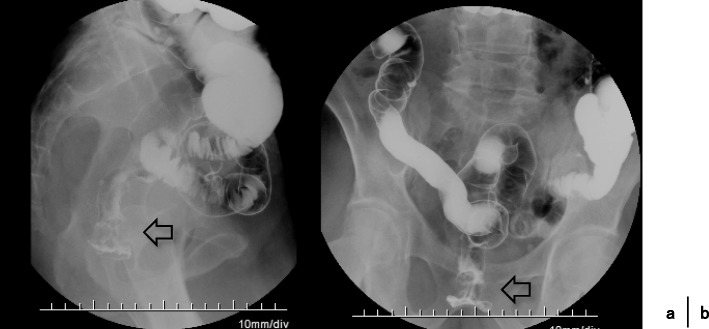
Barium enema showed the apple core sign at the rectum below the peritoneal reflection (arrow)

**Fig. 2:**
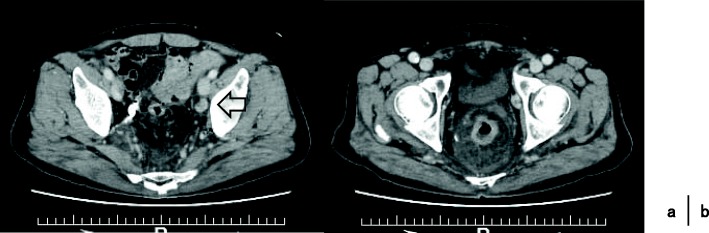
**a** The left-sided lateral lymph nodes were swollen (arrow). **b** Fat concentration was increased

**Fig. 3:**
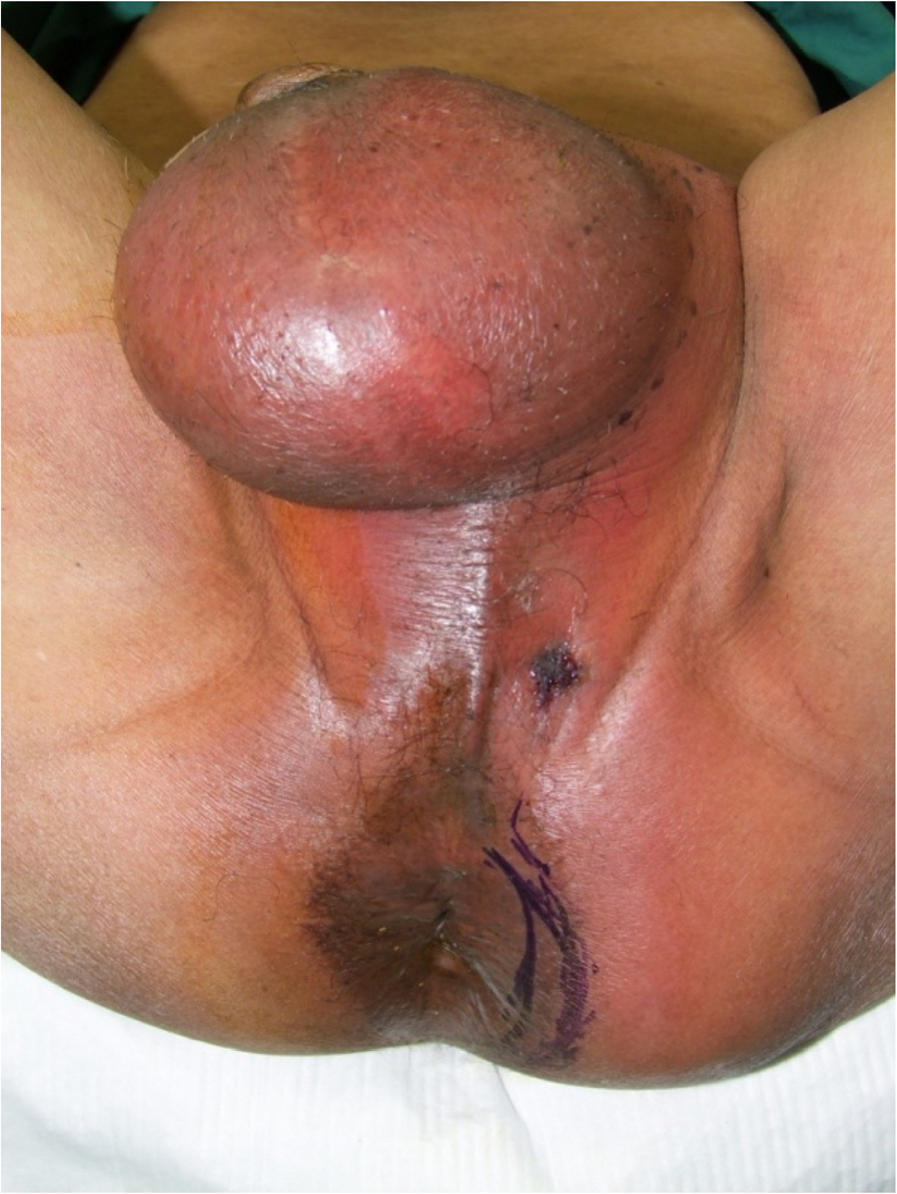
The perineum and scrotum was swollen

**Fig. 4:**
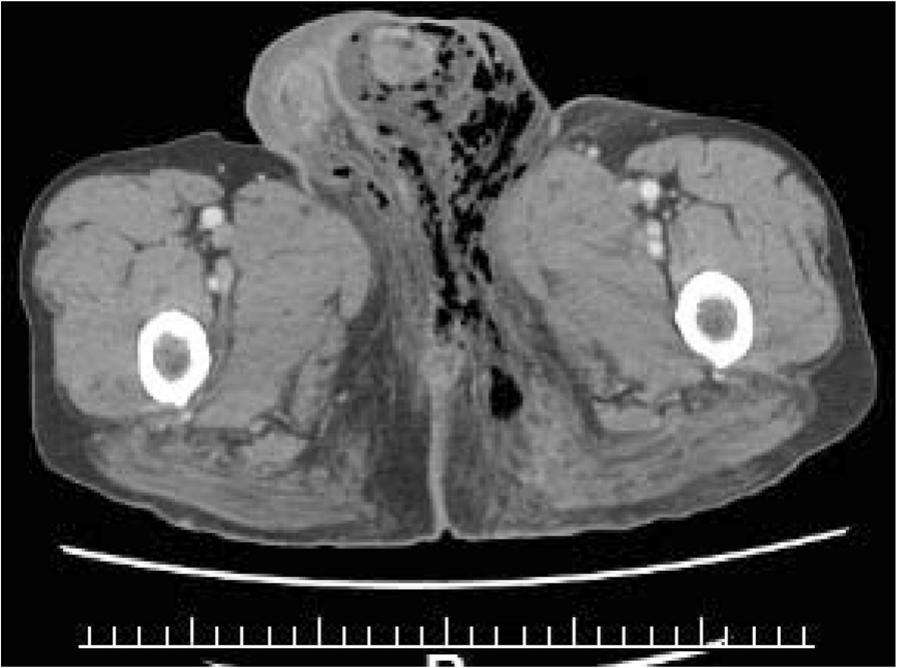
Extensive emphysema in the scrotum and gluteus maximus muscle

A 68-year-old Japanese man presented at our hospital for evaluation and treatment of rectal cancer. He had no history of rectal cancer. He had been diagnosed with rectal cancer using colonoscopy for screening of rectal bleeding; following biopsy, the tumor was confirmed to be moderately differentiated adenocarcinoma. Barium enema showed a filling defect at the rectum below the peritoneal reflection (Fig. 1). Contrast-enhanced CT (CECT) revealed swollen lateral lymph nodes (Fig. 2). We diagnosed him as having rectal cancer cT4aN1M0 clinical stageIIIa (UICC 8th). We decided to perform neoadjuvant chemotherapy for locally advanced rectal cancer for the purpose of the prevention of postoperative local recurrence. We performed CapeOX therapy 1 course until the RAS results were out. Since the RAS gene was confirmed as the wild-type, we changed the regimen and performed FOLFOX + cetuximab therapy 1 course to reduce the tumor size. The reason why we chose cetuximab is we can perform chemotherapy with cetuximab until just before surgery. On day 14, he visited the emergency room due to perineal pain. Physical findings showed BT 38.0 °C, HR 117/min, BP 79/57 mmHg, tenderness, redness, and swelling of the perineum and scrotum (Fig. 3). He had no tenderness of the abdomen. CECT revealed extensive emphysema in the scrotum and gluteus maximus muscle (Fig. 4). We diagnosed as Fournier’s gangrene caused by penetration of a rectal cancer. We performed debridement, left orchiectomy, transverse colostomy with double orifices. Abscess cultures yielded *Escherichia coli*. Intravenous antimicrobial treatment was administered using cefmetazole (1 g every 6 h).

**Fig. 5:**
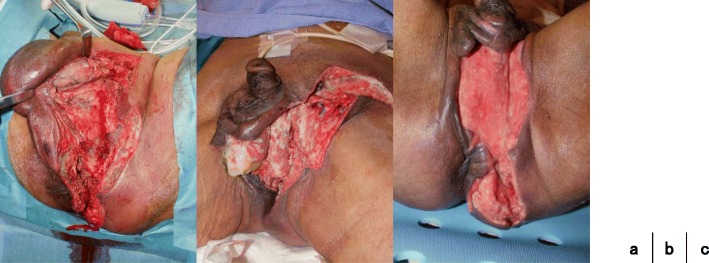
Debridement, left orchiectomy, transverse colostomy with double orifices (**a**). We cleaned the wound every day (**b**). The granulation tissue was formed (**c**)

**Fig. 6:**
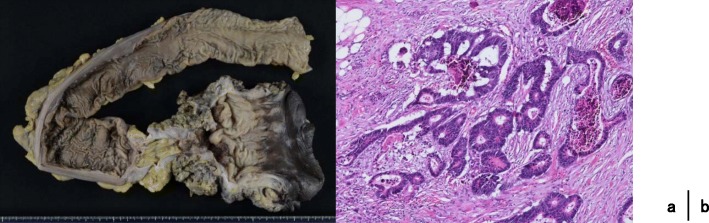
Resected specimen (**a**). Histological effect judgment was grade 1a (RECIST v1.1) (**b**)

After the first operation, we cleaned the wound every day. Because his right testis was in poor condition, we performed right orchiectomy under local anesthesia on postoperative day (POD) 7. Once the granulation tissue had formed (Fig. 5), we performed abdominoperineal resection (APR) and dissected the left lateral lymph nodes. on POD30 (Fig. 6). The amount of blood loss was 320 ml, and the operation time was 269 min. Pathological findings were type3, 35 × 30 mm, tub2, ly1, v2, ypT3N1a(263), and M0 Stage IIIa (UICC 8th). Histological grade was 1a (RECIST v1.1). The tumor left side wall was thin. Because the effect of chemotherapy was poor, we diagnosed as Fournier’s gangrene caused by penetration of rectal cancer.

**Fig. 7:**
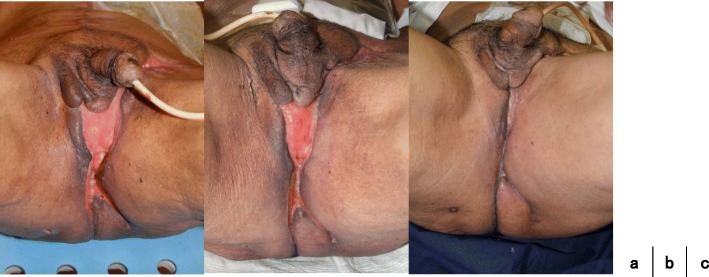
Cleaned the wound every day (**a**). At discharge (**b**). Healing (**c**)

After the second operation, we cleaned the wound every day. He left the hospital on POD59. On POD92, the wound was healed like that in the picture (Fig. 7). Since he wanted to leave the hospital, we showed him the treatment procedure for scar healing. We performed CapeOX therapy eight courses as adjuvant chemotherapy. The patient had no recurrence for 1 year and 2 months after the operation.

## Discussion

Fournier’s gangrene (FG) is a rare, life-threatening, necrotizing fasciitis that usually involves the perineal or genital areas. It was described by Baurienne in 1764 and formally named by Fournier in 1883 [2]. Prompt identification of this deadly disease is fundamental for appropriate treatment. Despite adequate treatment, the mortality rate is very high, reaching 20–35% [1].

Accepted predisposing factors for FG are poor perfusion (peripheral vascular disease), hypertension, renal insufficiency, trauma, diabetes mellitus, malnutrition, smoking, obesity, immunocompromised status, intravenous drug abuse, malignancy, and spinal cord injury [3, 4]. Old age is not a predisposing factor; however, elderly patients with poor self-care and poor nutritional status are more susceptible. Female to male ratio varies significantly. The lower incidence in women is ascribed to better drainage of the perineal region through vaginal secretions [5].

There are three development paths: (1) trauma surrounding the perineum, (2) progress along the muscle membrane surface of the infected urinary tract, and (3) infection of the perianal and retroperitoneum [4].

Treatment with broad-spectrum antibiotic coverage to include staphylococci, streptococci, Enterobacteriaceae, and anaerobes should be promptly initiated. However, aggressive surgical management remains the mainstay of treatment. This includes wide excision of all necrotic tissue down to bleeding tissues [6]. Oftentimes, repeated operative debridements may be needed to sufficiently debride all involved tissue.

Although perforation of rectal cancer after treatment with bevacizumab or radiation therapy has been well documented, reports of spontaneous perforation of rectal cancer presenting as FG are rare. There have been some reports on the onset of FG caused by penetration of a rectal cancer followed by neoadjuvant chemotherapy. In the world, 35 cases of FG caused by penetration of rectal cancer, including the present case, have been reported (Table 1). The median age in the Japanese group was 61.1 years with a male to female ratio of 31:4. Nine (25.7%) of 35 patients had diabetes mellitus (DM) as a comorbidity. DM was considered a risk factor for FG in the Japanese group as well. Debridement was performed in 32 of 35 patients. It is considered to be essential thorough debridement. Colostomy, including APR and total pelvic exenteration (TPE), was created in 30 of 35 patients. Only five patients were able to avoid a colostomy. With respect to colostomy for FG, this does not have just one point of view [7]. But we considered that colostomy was an effective treatment for FG caused by penetration of rectal cancer or anal canal cancer. Treatment for rectal cancer with FG was APR for 13 patients, TPE for five patients, and chemotherapy alone for six patients. With respect to outcome, seven (20%) patients were dead in the hospital. Seven (20%) patients were treated with preoperative chemotherapy.

**Table 1:** Clinical data of patients of Fournier’s gangrene caused by penetration of rectal cancer

Case	Author	Year	Age	Sex	DM	Origin	Histology	1st operation	2nd operation	3rd operation	Preoperative chemotherapy	Outcome
1	Futamura [8]	1995	56	M	−	Rb	tub1	I	D	APR	−	Alive (4 years and 7 months)
2	Nakao [9]	1999	51	M	+	Rab	tub1	D	C		−	Unknown
3	Fujisawa [10]	1999	75	M	−	Rb	Unknown	I	D		−	Death (6 days)
4	Saito [11]	2000	60	M	−	Rab	tub1	D	C	TPE + anaplasty	–	Alive (1 year)
5	Noriyuki [12]	2003	58	M	+	Rab	tub2	I	D + C		−	Alive (1 year and 2 months)
6	Shino[13]	2003	62	M	−	RbP	tub2	I	D + C	APR	−	Alive (3 months)
7	Enomoto[14]	2006	35	M	−	Rab	tub1	D	APR		−	Alive (3 months)
8	Moriwaki [15]	2007	30	M	–	Rab	tub2	I + C	APR	D + anaplasty	–	Death (11 months)
9	Kojima [16]	2007	56	M	+	Rb	tub2	D + C	TPE		−	Alive (4 months)
10	Morohashi [17]	2008	60	M	+	Rb	tub1	D	D + C		–	Death (2 months)
11	Ishibashi [18]	2009	80	F	−	Rb	Unknown	D	Anaplasty		−	Alive (2 months)
12	Suzuki [19]	2009	60s	M	−	Rb	tub1	D	D + C		–	Death (2 months)
13	Moslemi [20]	2009	48	M	Unknown	Unknown	Unknown	I + C			–	Alive (unknown)
14	Yamazaki [21]	2010	50s	M	−	Rb	tub2	D	APR	Anaplasty	−	Alive (1 month)
15	Onizuka [22]	2010	55	M	−	Rs-b	Unknown	D	D + C		–	Alive (1 year and 2 months)
16	Tanaka [23]	2010	52	F	+	Rb	muc	D	D + C	APR	−	Alive (5 months)
17	Matsuda [24]	2010	51	M	–	Unknown	Unknown	D	C		–	Death (10 months)
18	Matsuda [24]	2010	69	M	–	Unknown	Unknown	D + C			–	Alive (1 year and 10 months)
19	Watanabe [25]	2013	77	M	−	Rb-P	tub1	D + C			−	Death (70 days)
20	Takahashi [26]	2013	79	M	+	Rab	tub1	D + C	APR + anaplasty		–	Alive (unknown)
21	Momma [27]	2013	79	M	−	Rb-P	Unknown	I	D		+	Alive (1 year)
22	Kawagoe [28]	2014	72	M	−	Rb	Unknown	D	D + C		−	Alive (6 months)
23	Ishikawa [29]	2015	74	M	−	Rb	tub2	D	TPE		−	Alive (4 years 10 months)
24	Tsutsuyama [30]	2015	61	M	−	Rb	tub2	D + C	APR		−	Alive (3 months)
25	Abe [31]	2015	71	F	−	Rb	tub2	D + C	APR		−	Alive (3 years 6 months)
26	Tanaka [32]	2016	67	M	+	Rb	tub2	D + C	APR		−	Alive (1 year)
27	Kuroda [33]	2016	63	M	+	Rb	tub2	D + C	APR		−	Alive (2 years and 9 months)
28	Yoshino [34]	2016	65	M	Unknown	Unknown	Unknown	D + C	TPE + anaplasty		−	Alive (108 days)
29	Suzuki [35]	2017	64	M	+	RaRb	tub2	D	APR		−	Death (3 years and 10 months)
30	Koyama [36]	2017	66	M	−	Unknown	Unknown	I			+	Alive (12 days)
31	Koyama [36]	2017	63	M	Unknown	Unknown	muc	I			+	Alive (18 days)
32	Sawayama [37]	2017	66	M	Unknown	Unknown	Unknown	D	TPE + anaplasty		+	Alive (unknown)
33	Fukuhisa [38]	2017	73	M	Unknown	Unknown	Unknown	D + C			+	Alive (50 days)
34	Pittaka [39]	2018	24	F	Unknown	Unknown	Unknown	D			+	Alive (1 year)
35	Our case	2018	68	M	−	Rb	tub2	D+C	APR		+	Alive (1 year and 2 months)

*I* incision and drainage, *D* debridement, *C* colostomy, *APR* abdominoperineal resection, *TPE* total pelvic exentration

In our case, we promptly diagnosed as FG caused by penetration of a rectal cancer followed by neoadjuvant chemotherapy, and performed debridement and colostomy as soon as possible. After that, we performed APR and got a good prognosis.

The disease is life-threatening and has a high mortality rate. Therefore, prompt diagnosis and treatment (debridement and antibiotics) is recommended. Going forward, knowledge gained from this case will increase the opportunity to perform neoadjuvant chemotherapy for locally advanced rectal cancer. In medical treatment, we must put the possibility of Fournier’s gangrene in mind and treat as soon as possible.

## Conclusions

We reported the first case of Fournier’s gangrene caused by penetration of a rectal cancer followed by neoadjuvant chemotherapy with a review of literature.

## Abbreviations

APR: Abdominoperineal resection
CECT: Contrast-enhanced computed tomography
DM: Diabetes mellitus
FG: Fournier’s gangrene
POD: Post-operative day
TPE: Total pelvic exenteration
